# PERCEPTION OF NEIGHBORHOOD WALKABILITY INFLUENCES PHYSICAL THERAPISTS’ WALKING RECOMMENDATIONS FOR OLDER ADULTS

**DOI:** 10.1093/geroni/igae098.2956

**Published:** 2024-12-31

**Authors:** Jennifer Blackwood, Hannah Karczewski

**Affiliations:** University of Michigan, Ann Arbor, Michigan, United States; University of Michigan Flint, Flint, Michigan, United States

## Abstract

**Background/objectives:**

Neighborhood walkability is the extent to which built and social environments support walking. Walkability influences older adults’ participation in outdoor walking. Physical therapists (PTs) may identify factors that influence decisions about outdoor participation in walking. Current understanding of PTs’ knowledge and perceptions of walkability, and utilization of walkability assessments is not described and serves as the purpose of this study.

**Methods:**

A cross-sectional survey was sent via email to 5000 PTs nationwide with IRB approval. The 40-item survey covered the following: walking and walkability perceptions and assessments, perception of responsibilities, and demographics. Categorical variables were compared using Chi-square analyses.

**Results:**

After inclusion and exclusion criteria, a total of 122 outpatient PTs who worked in geriatrics were used for analysis. A significant difference was found between perceptions of whether PTs should assess walkability and whether they assess walkability (x2=78.7, p< 0.001). Components that influenced decisions to prescribe outdoor walking were prioritized in this order: availability (n=79, 64.8%) and maintenance (n=11, 9.0%) of sidewalks, crime (n=9, 7.4%), hills (n=7, 5.7%), aesthetics (n=6, 4.9%), and other (n=10, 8.2%). Objective measures were not used to assess walkability.

**Conclusion:**

Respondents had a significant difference between perceptions of their responsibility to assess walkability and whether they do assess walkability in older adults before prescribing walking. Built environment constructs were prioritized over the social environment. Significance/Implications: Assessment of walkability may allow PTs to identify barriers and make more informed recommendations concerning outdoor walking for older adults. Objective measures are available for PTs when prescribing outdoor walking.

